# Sarilumab plus methotrexate in patients with active rheumatoid arthritis and inadequate response to methotrexate: results of a randomized, placebo-controlled phase III trial in Japan

**DOI:** 10.1186/s13075-019-1856-4

**Published:** 2019-03-20

**Authors:** Yoshiya Tanaka, Kazuteru Wada, Yoshinori Takahashi, Owen Hagino, Hubert van Hoogstraten, Neil M. H. Graham, Hideto Kameda

**Affiliations:** 1 The First Department of Internal Medicine, School of Medicine, University of Occupational and Environmental Health, Japan, 1-1 Iseigaoka, Yahata-nishi-ku, Kitakyushu, 807-8555 Japan; 2 Sanofi K.K, Opera City Tower 3-20-2 Nishi-Shinjuku, Shinjuku-ku, Tokyo, 163-1488 Japan; 3 Sanofi, 55 Corporate Drive, Bridgewater, NJ 08807 USA; 4 Sanofi-Genzyme, 500 Kendall St, Cambridge, MA 02142 USA; 5 Regeneron Pharmaceuticals, Inc., 777 Old Saw Mill River Road, Tarrytown, NY 10591 USA; 6 Division of Rheumatology, Department of Internal Medicine, School of Medicine, Toho University, 2-22-36 Ohashi, Meguro-ku, Tokyo, 153-8515 Japan

**Keywords:** Rheumatoid arthritis, Sarilumab, Japan, Phase III, Antibody, Anti-IL-6 receptor, MTX-IR

## Abstract

**Background:**

Sarilumab is a human immunoglobulin G1 anti-interleukin-6 (IL-6) receptor monoclonal antibody that blocks IL-6 from binding to membrane-bound and soluble IL-6 receptor α. This bridging study assessed the efficacy and safety of sarilumab + methotrexate (MTX) in Japanese patients with active rheumatoid arthritis (RA) and inadequate response to MTX (MTX-IR).

**Methods:**

In this phase III study, 243 patients were randomized 2:2:1:1 to receive subcutaneous sarilumab 150 mg every 2 weeks (q2w), sarilumab 200 mg q2w, placebo switching to sarilumab 150 mg q2w + MTX at 24 weeks, or placebo switching to sarilumab 200 mg q2w at 24 weeks, all in combination with MTX, for a total of 52 weeks (double-blind, placebo-controlled 24-week period followed by a single-blind 28-week extension). The primary endpoint was the proportion of patients achieving American College of Rheumatology 20% improvement criteria (ACR20) responses at week 24.

**Results:**

ACR20 response rates at week 24 were 67.9%, 57.5%, and 14.8% for sarilumab 150 mg, sarilumab 200 mg, and placebo, respectively. Serious treatment-emergent adverse events were reported by 9.9%, 6.3%, 0%, and 13.3% of patients in the sarilumab 150 mg, sarilumab 200 mg, placebo to sarilumab 150 mg, and placebo to sarilumab 200 mg groups, respectively. No deaths occurred. The incidence of infections ranged from 52.5 to 67.9%, with five serious infections for the sarilumab 150 mg group and one for the group switched from placebo to 200 mg sarilumab. Absolute neutrophil count < 1.0 Giga/l occurred in 13.6% and 7.5% of patients in the sarilumab 150 and 200 mg groups, respectively, and was not associated with infection.

**Conclusions:**

In Japanese MTX-IR RA patients treated with sarilumab (150 and 200 mg q2w) in combination with MTX, sustained clinical efficacy was shown by significant improvement in signs, symptoms, and physical function; bridging between this and a previous global study was achieved. At week 52, the safety profiles of both doses of sarilumab were generally similar, as previously observed and as expected based on IL-6 class.

**Trial registration:**

ClinicalTrials.gov, NCT02293902. Registered on 19 November 2014.

**Electronic supplementary material:**

The online version of this article (10.1186/s13075-019-1856-4) contains supplementary material, which is available to authorized users.

## Background

Rheumatoid arthritis (RA) is a chronic and debilitating autoimmune disease characterized by persistent synovitis and systemic inflammation, ultimately resulting in joint damage, disability, decreased quality of life, and cardiovascular and other comorbidities [1]. Disease-modifying antirheumatic drugs (DMARDs) are the key therapeutic agents and include conventional synthetic DMARDs (csDMARDs), of which methotrexate (MTX) is the anchor drug, as well as biological and targeted synthetic DMARDs targeting tumor necrosis factor (TNF) α, interleukin-6 (IL-6) receptor (IL-6R), T cell costimulation, B cells (CD20), and Janus kinase inhibitors. Recent guidelines for the management of RA recommend rapid attainment of sustained remission or low disease activity in every patient [2]. However, many patients do not respond sufficiently to current therapies [2].

IL-6 is a key cytokine in the pathogenesis of RA [3]. Sarilumab is a human immunoglobulin G1 anti-IL-6R monoclonal antibody that blocks IL-6 from binding to both membrane-bound and soluble IL-6Rα [4]. The efficacy and safety of sarilumab added to MTX has been investigated in the double-blind, placebo-controlled, dose-ranging, and confirmatory MOBILITY study in non-Japanese patients with active RA who were inadequate responders to MTX therapy [5]. In MOBILITY, both 150 mg every 2 weeks (q2w) and 200 mg q2w showed sustained efficacy, with significant improvements in the signs and symptoms of RA, physical function, and radiographic outcomes. Although the MOBILITY study was not powered to detect any difference between doses of sarilumab, substantially greater inhibition of structural damage progression (as shown by radiography) was observed with the 200-mg q2w dose compared with the 150-mg q2w dose. The safety profile was consistent with previous studies [6, 7] and with effects of IL-6 signaling blockade, a higher incidence of infections, elevated alanine aminotransferase (ALT) and total serum cholesterol, and decreased neutrophil count (but not associated with the occurrence of infections) with sarilumab compared with placebo.

The efficacy and safety of sarilumab monotherapy compared with adalimumab monotherapy has been evaluated in the MONARCH study over 24 weeks in non-Japanese patients with active RA with intolerance or inadequate response to MTX therapy [8]. In MONARCH, sarilumab 200 mg q2w was superior to adalimumab 40 mg q2w in the primary endpoint of change from baseline in Disease Activity Score 28-joint count (DAS28) erythrocyte sedimentation rate (ESR). Sarilumab-treated patients also achieved significantly higher American College of Rheumatology (ACR) 20%/50%/70% improvement criteria (ACR20/50/70) response rates and showed significantly greater improvement in Health Assessment Questionnaire-Disability Index (HAQ-DI), and more patients receiving sarilumab achieved Clinical Disease Activity Index (CDAI) ≤ 2.8 than those receiving adalimumab. Safety profiles, including rates of infection, were similar for sarilumab and adalimumab.

The efficacy and safety of sarilumab in combination with csDMARDs was investigated in the TARGET study in patients with an inadequate response or intolerance to anti-TNF therapy [9]. In TARGET, sarilumab 150 and 200 mg q2w + csDMARDs improved the signs and symptoms of RA and physical function in patients with an inadequate response or intolerance to anti-TNF agents; thus, results were similar to those from MOBILITY, although radiographic progression was not assessed in TARGET. Safety data were consistent with the effects of IL-6 signaling blockade and the known safety profile of sarilumab.

In the phase III KAKEHASI study, we evaluated the efficacy and safety of subcutaneous (SC) sarilumab added to MTX in patients with RA with inadequate response to MTX in Japan. Positive efficacy results for both dose regimens, as shown by statistically significant differences from placebo in the ACR20 response rates at week 24, would permit bridging between the KAKEHASI study and MOBILITY findings in non-Japanese patients.

## Methods

### Study design

The KAKEHASI trial (NCT02293902) was a multicenter, randomized, 52-week, parallel-group study with a 24-week double-blind placebo-controlled period followed by a 28-week single-blind uncontrolled extension period, during which patients in the placebo arm were switched to sarilumab. Patients with an inadequate response to MTX were randomized (2:2:1:1) to receive SC injections of sarilumab or placebo in one of the following four regimens, with MTX as background therapy: sarilumab 150 mg (SC) q2w; sarilumab 200 mg (SC) q2w; placebo (SC) q2w, switching to sarilumab 150 mg (SC) q2w at week 24; or placebo (SC) q2w, switching to sarilumab 200 mg (SC) q2w at week 24. Patients with an inadequate response by week 16, defined as < 20% improvement from baseline on two consecutive visits (at least 4 weeks apart) in either tender joint count (TJC) or swollen joint count (SJC), or with clear lack of efficacy based on investigator judgment, were proposed for rescue with sarilumab 200 mg q2w.

Randomization was performed centrally via an interactive voice or interactive web response system, with allocation stratified by previous biologics use (yes/no) and body weight (< 55 kg, ≥ 55 kg). Sarilumab and matching placebo were provided in identical glass prefilled syringes. Investigators and site staff were blinded, with no access to randomization information (the exception being for code-breaking if an adverse event (AE) occurred for which knowledge of the investigational product was needed to treat the patient). The number of swollen and tender joints was evaluated by a blinded assessor who had no access to any patient data, including previous joint assessments, during the study.

The study was performed in accordance with applicable laws and guidelines, including the Declaration of Helsinki and the International Council for Harmonisation guidelines for Good Clinical Practice. The protocol and amendments were approved by independent ethics committees and/or institutional review boards and written informed consent was obtained from all participants prior to the conduct of any study-related procedures.

### Patient population

Patients had to be aged 20–75 years, fulfilling the ACR/European League Against Rheumatism (EULAR) 2010 RA classification criteria, and have an ACR Class I-III functional status (1991 revised criteria [10]). Patients were included if they had moderately to severely active RA (defined as ≥ 8 of 68 tender joints and ≥ 6 of 66 swollen joints, and high-sensitivity (hs) CRP ≥ 0.6 mg/dl), with ≥ 3 months’ disease duration despite continuous treatment with MTX for at least 12 weeks at a stable dose (6–16 mg/week) at the time point ≥ 6 weeks prior to screening. Patients were excluded if they had uncontrolled concomitant diseases, severe systemic RA, other autoimmune or inflammatory systemic or localized joint diseases, current/recurrent infections, or past history of nonresponse to prior therapy with a TNF antagonist or a biologic treatment.

### Efficacy assessments

The primary endpoint was ACR20 response at week 24. Exploratory efficacy endpoints included the following: ACR20 at weeks 12 and 52; ACR50/70 at weeks 12, 24, and 52; mean change from baseline in DAS28-CRP at weeks 12, 24, and 52; a DAS28-CRP score of < 2.6 at weeks 12, 24, and 52; mean change from baseline in HAQ-DI at weeks 12, 16, 24, and 52; mean change from baseline in Simplified Disease Activity Index (SDAI) at weeks 12, 24, and 52; mean change from baseline in CDAI at weeks 12, 24, and 52; and SDAI ≤ 3.3 and CDAI ≤ 2.8 at weeks 12, 24, and 52. Post hoc analysis was performed to assess the proportion of patients exhibiting total suppression of CRP (hs-CRP at or below lower limit of detection (0.02 mg/dl)) in each group.

The study was not powered to demonstrate a difference between sarilumab 150 mg q2w + MTX or sarilumab 200 mg q2w + MTX; therefore, to further investigate efficacy differences between the two doses, a post hoc analysis was performed in which efficacy results over the first 12 weeks after patients switched from placebo + MTX to sarilumab at week 24 were added to results from the patients initially treated with the 150- and 200-mg doses.

### Safety assessments

Safety assessments encompassed AEs (including treatment-emergent AEs (TEAEs), serious TEAEs, and AEs of special interest), laboratory safety variables, vital signs, physical examination, and electrocardiograms (ECGs). For patients rescued before week 52, only the safety data collected before rescue were presented for each treatment group.

### Statistical analysis

A sample size of 80 patients per treatment group was calculated to provide more than 90% power for each pair-wise comparison between placebo and the two sarilumab doses based on Fisher’s exact test with alpha = 0.025 (two-sided), assuming ACR20 response rates at week 24 of 33.4% and 62% in the placebo and active dose groups, respectively. The two placebo groups were combined for the statistical analyses at 24 weeks. The primary efficacy population was the modified intent-to-treat (mITT) population, which included all randomized patients who received at least one dose of study medication and had an evaluable primary endpoint, irrespective of compliance with the study protocol and procedures. Patients were analyzed according to the treatment to which they were randomized. Efficacy data collected after treatment discontinuation or rescue were set to missing and no imputation was performed. Patients were considered nonresponders from the time they started rescue medication or discontinued study medication.

The safety population included all patients who received at least one dose or a partial dose of study medication. Safety data were analyzed as observed according to the treatment actually received.

The primary endpoint of ACR20 response at week 24 was analyzed as the proportion of patients who achieved ACR20 at week 24 using the two-sided Cochran-Mantel-Haenszel test, stratified by prior use of biologic agents and by weight (< 55 kg, ≥ 55 kg) at screening.

Exploratory efficacy variables were assessed in the mITT patient populations. Binary exploratory efficacy variables were analyzed up to week 24 by the two-sided Cochran-Mantel-Haenszel test, stratified by prior use of biologic agents and by weight (< 55 kg, ≥ 55 kg) at screening to assess treatment differences in the following endpoints: ACR20 at week 12; ACR50 at weeks 12 and 24; ACR70 at weeks 12 and 24; DAS28-CRP < 2.6 at weeks 12 and 24; HAQ-DI response (≥ 0.3 and ≥ 0.22 units of improvement in change from baseline) at weeks 12, 16, and 24; CDAI ≤ 2.8 and SDAI ≤ 3.3 at weeks 12 and 24. The analyses of binary exploratory efficacy variables at each visit and after week 24 were essentially descriptive. Continuous exploratory efficacy endpoints were analyzed up to week 24 with a mixed-model repeated measures approach.

All safety analyses were performed on the safety population and included AEs and serious AEs coded using the Medical Dictionary for Regulatory Activities (MedDRA) version 17.1, and summary statistics for laboratory values, vital signs, and ECGs.

## Results

### Patients

**Fig. 1:**
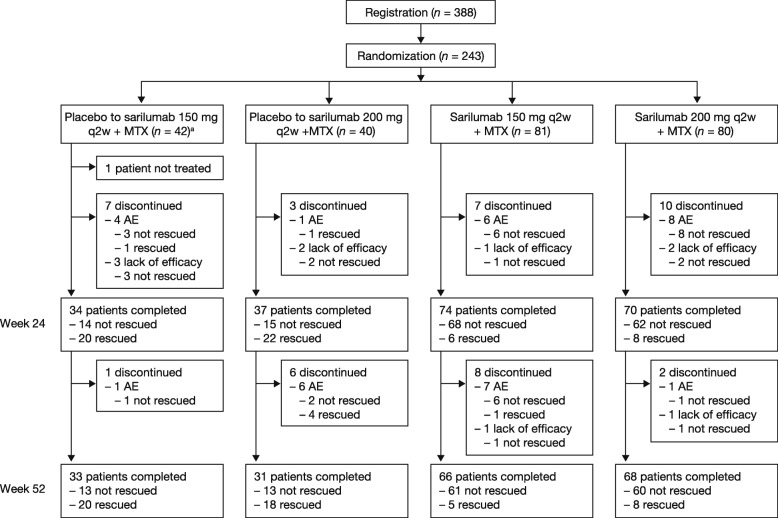
Patient disposition. ^a^Out of 243 patients, 1 patient in the placebo to sarilumab 150 mg group was not treated; therefore, 242 patients were included in the modified intent-to-treat population for the KAKEHASI study. AE adverse event, MTX methotrexate, q2w every 2 weeks

In total, 243 patients were randomized to receive sarilumab 150 mg q2w (*n =* 81), sarilumab 200 mg q2w (*n =* 80), placebo followed by sarilumab 150 mg q2w (*n =* 42), or placebo followed by sarilumab 200 mg q2w (*n =* 40) in 95 sites in Japan (Fig. 1). One patient in the placebo to sarilumab 150 mg group was not treated due to meeting an exclusion criterion; this patient was excluded from all analysis populations. Of the 242 treated patients, 198 (81.8%) completed the 52-week treatment period. The first patient was enrolled in November 2014 and the last patient completed the trial in October 2016. More patients in the placebo groups (21/42 (50.0%) placebo to sarilumab 150 mg and 23/40 (57.5%) placebo to sarilumab 200 mg) than in the sarilumab 150 mg (6/81 (7.4%)) or sarilumab 200 mg group (8/80 (10.0%)) received rescue therapy up to week 24. One patient in each of the placebo groups discontinued rescue therapy before week 24 due to an AE. The proportion of patients who discontinued treatment was similar across the groups (8/42 (19.0%), 9/40 (22.5%), 15/81 (18.5%), and 12/80 (15.0%) in the placebo to sarilumab 150 mg, placebo to sarilumab 200 mg, sarilumab 150 mg, and sarilumab 200 mg groups, respectively).

Baseline demographics and disease characteristics were generally well balanced between the treatment groups (Table 1).

**Table 1:** Demographics and patient characteristics at baseline (randomized population)

	Sarilumab			
	Placebo to 150 mg q2w + MTX(*n* = 42)	Placebo to 200 mg q2w + MTX(*n* = 40)	150 mg q2w + MTX(*n* = 81)	200 mg q2w + MTX(*n* = 80)
Age, mean (SD) years	51.9 (11.0)	55.0 (11.9)	56.1 (9.5)	55.3 (11.0)
Female/male, %	81.0/19.0	77.5/22.5	77.8/22.2	76.3/23.8
Weight, mean (SD) kg	54.5 (11.9)	58.7 (12.3)	56.6 (12.4)	56.7 (10.9)
Race, %				
Asian	100	100	100	100
MTX dosage, mean (SD) mg/week	9.4 (3.2)	10.4 (3.2)	10.1 (3.0)	10.1 (3.0)
Prior biologic DMARD use, *n* (%)	16 (38.1)	7 (17.5)	28 (34.6)	22 (27.5)
Baseline corticosteroids, *n* (%)	22 (52.4)	17 (42.5)	42 (51.9)	46 (57.5)
Duration of RA, mean (range) years	7.6 (0.3–43.1)	8.8 (0.3–35.2)	7.0 (0.3–37.5)	8.3 (0.3–33.6)
Seropositive for rheumatoid factor, *n* (%)	32 (76.2)	24 (61.5)^a^	62 (76.5)	60 (75.0)
Anti-CCP antibody positive, *n* (%)	36 (85.7)	32 (82.1)^a^	71 (87.7)	71 (88.8)
DAS28-CRP, mean (SD)	5.6 (0.8)	5.3 (1.0)	5.7 (1.0)	5.4 (0.9)
TJC, mean (SD)	18.9 (10.2)	17.2 (10.4)	19.3 (12.1)	17.9 (12.4)
SJC, mean (SD)	15.1 (7.6)	14.1 (8.7)	16.1 (9.0)	14.4 (9.7)
CRP, mean (SD) mg/l	23.7 (19.9)	21.0 (22.8)	22.9 (19.9)	23.1 (20.6)
SDAI, mean (SD)	36.7 (10.2)	34.3 (12.1)	38.2 (13.2)	35.2 (12.9)
CDAI, mean (SD)	34.4 (9.5)	31.9 (11.2)	35.9 (12.6)	32.9 (11.9)
HAQ-DI score, mean (SD)	1.1 (0.6)	1.0 (0.7)	1.2 (0.7)	1.1 (0.7)

^a^*n* = 39

*CCP* cyclic citrullinated peptide, *CDAI* Clinical Disease Activity Index, *CRP* C-reactive protein, *DAS28* Disease Activity Score 28-joint count, *DMARD* disease-modifying antirheumatic drug, *HAQ-DI* Health Assessment Questionnaire-Disability Index, *MTX* methotrexate, *q2w* every 2 weeks, *RA* rheumatoid arthritis, *SD* standard deviation, *SDAI* Simplified Disease Activity Index, *SJC* swollen joint count, *TJC* tender joint count

### Efficacy

**Fig. 2:**
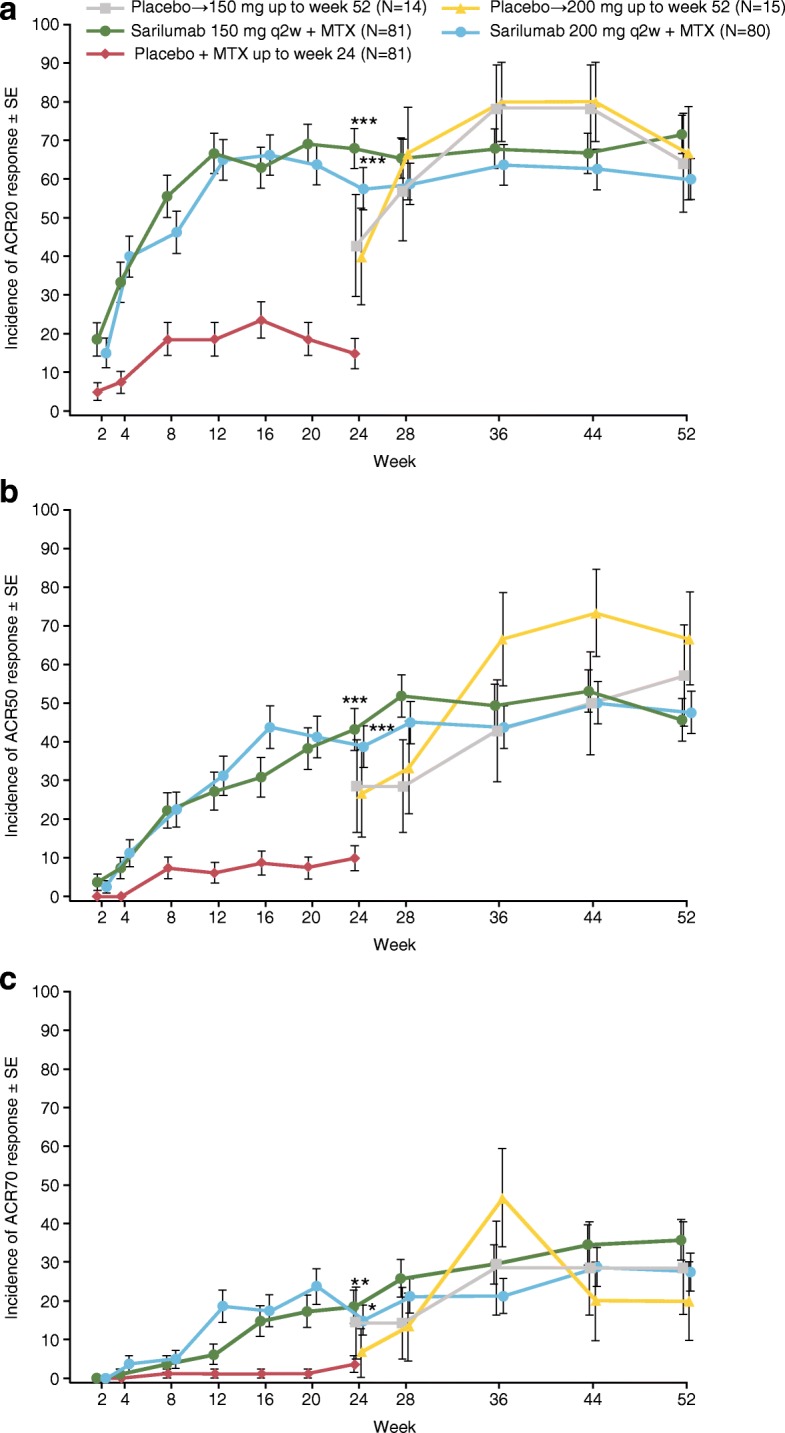
Proportion of patients who achieved **a** ACR20, **b** ACR50, and **c** ACR70 improvement responses at weeks 12, 24, and 52. *p < 0.05, **p < 0.01, ***p < 0.001 Cochran-Mantel-Haenszel test stratified by prior biologic use and weight (< 55 kg, ≥ 55 kg) versus placebo at week 24. Patients were considered nonresponders from the time they started rescue medication or discontinued study medication. Primary endpoint was the proportion of patients achieving ACR20 response at week 24. ACR20/50/70 American College of Rheumatology 20%/50%/70% improvement criteria, MTX methotrexate, q2w every 2 weeks, SE standard error

The primary efficacy analysis at week 24 showed that ACR20 response rates in both sarilumab dose groups were superior to placebo (55/81 (67.9%), 46/80 (57.5%), and 12/81 (14.8%) for sarilumab 150 mg, sarilumab 200 mg, and placebo, respectively; *p* <  0.001 for each sarilumab dose vs placebo) (Table 2). The ACR20 response was maintained by sarilumab throughout the duration of the study, with response rates of 58/81 (71.6%) and 48/80 (60.0%) for sarilumab 150 and 200 mg, respectively, at week 52. For those who switched from placebo to sarilumab, the majority of patients achieved an ACR20 response at week 52 (9/14 (64.3%) and 10/15 (66.7%) in the placebo to sarilumab 150 and 200 mg groups, respectively) (Fig. 2a).

**Table 2:** Efficacy results (mITT population)

	Sarilumab			
	Placebo to 150 mg q2w + MTX (*n* = 41 (*n* = 14 at week 52))^a^	Placebo to 200 mg q2w + MTX (*n* = 40 (*n* = 15 at week 52))^a^	150 mg q2w + MTX (*n* = 81)	200 mg q2w + MTX (*n* = 80)
Signs and symptoms				
ACR20 response, *n* (%)				
At week 12	15 (18.5)		54 (66.7)^***^	52 (65.0)^***^
At week 24	12 (14.8)		55 (67.9)^***^	46 (57.5)^***^
At week 52	9 (64.3)	10 (66.7)	58 (71.6)	48 (60.0)
ACR50 response, *n* (%)				
At week 12	5 (6.2)		22 (27.2)^***^	25 (31.3)^***^
At week 24	8 (9.9)		35 (43.2)^***^	31 (38.8)^***^
At week 52	8 (57.1)	10 (66.7)	37 (45.7)	38 (47.5)
ACR70 response, *n* (%)				
At week 12	1 (1.2)		5 (6.2)	15 (18.8)^***^
At week 24	3 (3.7)		15 (18.5)^**^	12 (15.0)^*^
At week 52	4 (28.6)	3 (20.0)	29 (35.8)	22 (27.5)
ACR components, mean (SD) change from baseline at week 24				
Tender joint count	− 9.1 (10.2)		− 13.4 (9.9)	− 12.4 (11.3)
Swollen joint count	− 7.2 (6.7)		− 10.6 (8.1)	− 9.5 (9.1)
Pain VAS	− 22.9 (27.7)		− 36.5 (23.4)	− 30.2 (23.3)
Physician global VAS	− 26.8 (18.4)		− 41.8 (21.6)	− 43.9 (19.4)
Patient global VAS	− 18.3 (22.6)		− 32.4 (21.0)	− 30.6 (21.9)
HAQ-DI	− 0.3 (0.4)		− 0.5 (0.5)	− 0.6 (0.5)
CRP, mg/l	− 1.7 (12.2)		− 21.1 (19.5)	− 21.3 (18.0)
DAS28-CRP response, mean (SD) change from baseline				
At week 12	− 0.8 (1.1)		− 2.3 (1.1)^***^	− 2.3 (1.2)^***^
At week 24	− 1.5 (1.2)		− 2.8 (1.0)^***^	− 2.8 (1.1)^***^
At week 52	− 3.1 (1.2)	− 2.9 (1.2)	− 3.2 (1.2)	− 3.2 (1.1)
DAS28-CRP < 2.6, *n* (%)				
At week 12	3 (3.7)		21 (25.9)^***^	27 (33.8)^***^
At week 24	6 (7.4)		29 (35.8)^***^	32 (40.0)^***^
At week 52	7 (50.0)	9 (60.0)	41 (50.6)	43 (53.8)
SDAI, mean (SD) change from baseline				
At week 12	− 8.9 (12.0)		− 20.7 (11.0)^***^	− 18.9 (11.6)^***^
At week 24	− 16.0 (11.6)		− 25.2 (11.6)^***^	− 23.8 (11.3)^***^
At week 52	− 29.6 (9.9)	− 23.4 (12.4)	− 29.4 (13.6)	− 26.9 (11.5)
SDAI ≤ 3.3, *n* (%)				
At week 12	0		2 (2.5)	7 (8.8)^**^
At week 24	1 (1.2)		5 (6.2)	10 (12.5)^**^
At week 52	2 (14.3)	1 (6.7)	19 (23.5)	18 (22.5)
CDAI, mean (SD) change from baseline				
At week 12	− 8.7 (11.4)		− 18.8 (10.6)^***^	− 16.8 (10.9)^***^
At week 24	− 15.7 (11.1)		− 23.1 (11.2)^***^	− 21.7 (10.7)^***^
At week 52	− 28.4 (9.7)	− 21.1 (11.4)	− 27.2 (13.1)	− 24.8 (10.8)
CDAI ≤ 2.8, *n* (%)				
At week 12	0		1 (1.2)	5 (6.3)^*^
At week 24	1 (1.2)		5 (6.2)	8 (10.0)^*^
At week 52	1 (7.1)	0	17 (21.0)	15 (18.8)
Physical function				
HAQ-DI, mean (SD) change from baseline				
At week 12	− 0.1 (0.3)		− 0.4 (0.5)^***^	− 0.4 (0.5)^***^
At week 24	− 0.3 (0.4)		− 0.5 (0.5)^***^	− 0.6 (0.5)^***^
At week 52	− 0.7 (0.6)	− 0.5 (0.3)	− 0.6 (0.6)	− 0.6 (0.6)
HAQ-DI response (MCID ≥ 0.3), *n* (%)				
At week 12	19 (23.5)		39 (48.1)^**^	38 (47.5)^**^
At week 16	19 (23.5)		37 (45.7)^**^	37 (46.3)^**^
At week 24	10 (12.3)		39 (48.1)^***^	39 (48.8)^***^
At week 52	9 (64.3)	8 (53.3)	46 (56.8)	43 (53.8)

*p <  0.05; **p < 0.01; ***p < 0.001

^*a*^ Data for combined placebo groups (*n*=81) shown at weeks 12, 16 and 24. ACR American College of Rheumatology, *ACR20/50/70* American College of Rheumatology 20%/50%/70% improvement criteria, *CDAI* Clinical Disease Activity Index, *CRP* C-reactive protein, *DAS28* Disease Activity Score 28-joint count, *HAQ-DI* Health Assessment Questionnaire-Disability Index, *MCID* minimum clinically important difference, *mITT* modified intent-to-treat, *MTX* methotrexate, *q2w* every 2 weeks, *SDAI* Simplified Disease Activity Index, *SD* standard deviation, *SJC* swollen joint count, *TJC* tender joint count, *VAS* visual analog scale

The results of analyses of exploratory efficacy endpoints were consistent with the primary analysis (Table 2). ACR50 and ACR70 responses were similar in both sarilumab dose groups (Fig. 2b, c). Patients in both sarilumab dose groups reported similar improvement in all ACR component scores at week 52, which were generally consistent with the results at week 24 (Table 2).

Mean changes from baseline at week 52 in the exploratory parameters DAS28-CRP, HAQ-DI, SDAI, and CDAI were consistent with those of week 24 (Table 2). For both groups switching to sarilumab from placebo at week 24, the exploratory efficacy parameters showed improvements in measures of clinical response at week 52 (Table 2). The incidence of DAS28-CRP < 2.6 at week 24 was 29/81 (35.8%) in the sarilumab 150-mg dose group and 32/80 (40.0%) in the sarilumab 200-mg dose group compared with 41/81 (50.6%) in the sarilumab 150-mg dose group and 43/80 (53.8%) in the sarilumab 200-mg dose group at week 52. For the groups switching to sarilumab from placebo, the incidence was 7/14 (50.0%) in the placebo to sarilumab 150 mg group and 9/15 (60.0%) in the placebo to 200 mg group at week 52 (Table 2).

The HAQ-DI response rates (HAQ-DI ≥ 0.3 units of improvement) were similar in both sarilumab dose groups and consistent with the week 24 results (39/81 (48.1%) in the sarilumab 150-mg dose group and 39/80 (48.8%) in the sarilumab 200-mg dose group at week 24). For the groups switching to sarilumab from placebo at week 24, the HAQ-DI response rates were 9/14 (64.3%) in the placebo to sarilumab 150 mg group and 8/15 (53.3%) in the placebo to sarilumab 200 mg group at week 52 (Table 2).

The proportion of patients with SDAI ≤ 3.3 at week 24 was 5/81 (6.2%) in the sarilumab 150 mg group and 10/80 (12.5%) in the sarilumab 200 mg group, compared with 19/81 (23.5%) and 18/80 (22.5%), respectively, at week 52. For the groups originally receiving sarilumab, the proportion of patients was 2/14 (14.3%) in the placebo to sarilumab 150 mg group and 1/15 (6.7%) in the placebo to sarilumab group (Table 2). The incidence of CDAI ≤ 2.8 at week 24 was 5/81 (6.2%) in the sarilumab 150 mg group and 8/80 (10.0%) in the sarilumab 200 mg group, compared with 17/81 (21.0%) in the sarilumab 150 mg group and 15/80 (18.8%) in the sarilumab 200 mg group at week 52. For groups switching to sarilumab from placebo, the incidence was 1/14 (7.1%) in the placebo to sarilumab 150 mg group and 0 in the placebo to sarilumab 200 mg group at week 52 (Table 2).

In the post hoc analysis, efficacy results over the first 12 weeks after patients (*n =* 29) switched from placebo + MTX to sarilumab at week 24 were added to the results from the patients initially treated with the 150- and 200-mg dose regimens (*n =* 161). These analyses showed that, with the addition of a few more patients in each dose group (14 additional patients in the 150 mg group and 15 additional patients in the 200 mg group), response rates for the 200-mg dose regimen were generally numerically higher than those for the 150-mg q2w dose early (weeks 4 and 12) in the course of treatment (see Additional file 1: Table S1).

**Fig. 3:**
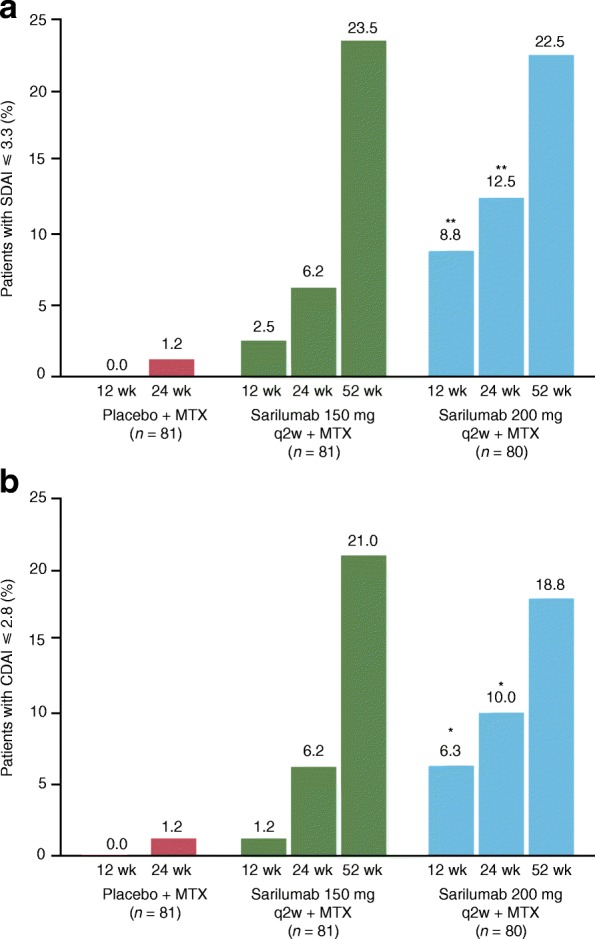
Proportion of patients with **a** SDAI ≤ 3.3, and **b** CDAI ≤ 2.8. *p < 0.05; **p < 0.01 (vs placebo + MTX group). Two-sided Cochran-Mantel-Haenszel test. CDAI Clinical Disease Activity Index, MTX methotrexate, q2w every 2 weeks, SDAI, Simplified Disease Activity Index

Analysis of 12-week data showed that a greater percentage of patients had better control of the signs and symptoms of RA (ACR50 and ACR70) and reduction of disease activity (DAS28-CRP < 2.6, SDAI ≤ 3.3, and CDAI ≤ 2.8) with sarilumab 200 mg + MTX compared with sarilumab 150 mg + MTX). A numerically higher proportion of patients achieved SDAI ≤ 3.3 and CDAI ≤ 2.8 earlier in the 200 mg group than in the 150 mg group (Table 2, Fig. 3).

**Fig. 4:**
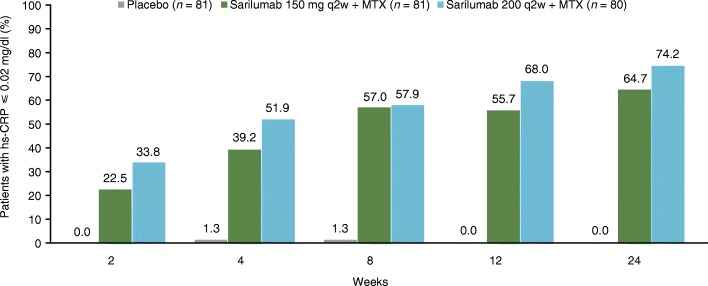
Proportion of patients with CRP level ≤ 0.02 mg/dl (post hoc analysis). CRP C-reactive protein, hs high-sensitivity, MTX methotrexate, q2w every 2 weeks

Post hoc analysis showed that from week 2 onwards, a numerically higher proportion of patients in the sarilumab 200 mg group exhibited total suppression of CRP (hs-CRP at or below lower limit of detection (0.02 mg/dl)) than in the 150 mg group (Fig. 4).

### Safety

The duration of study treatment during the 52-week treatment period was comparable within both sarilumab dose groups (mean 311 and 298 days for 150 and 200 mg, respectively) and within both placebo to sarilumab groups (mean 183 and 175 days for placebo to 150 and 200 mg, respectively).

A summary of AEs and the most common TEAEs is shown in Table 3. When compared with placebo during the double-blind period, the incidences of TEAEs and TEAEs leading to discontinuation were generally similar in both sarilumab groups and numerically higher than in the placebo group. There were no deaths. The two groups originally receiving sarilumab and the two groups switching to sarilumab from placebo had comparable incidences of TEAEs and TEAEs leading to discontinuation.

**Table 3:** Summary of treatment-emergent AEs in the safety population and most common treatment-emergent AEs

	Placebo-controlled period Sarilumab			Non-placebo-controlled period Sarilumab			
	Placebo + MTX (*n* = 81)	150 mg q2w + MTX (*n* = 81)	200 mg q2w + MTX (*n* = 80)	Placebo + MTX to sarilumab 150 mg q2w + MTX (*n* = 14)^a^	Placebo + MTX to sarilumab 200 mg q2w + MTX (*n* = 15)^a^	150 mg q2w + MTX (*n* = 81)	200 mg q2w + MTX (*n* = 80)
AEs	49 (60.5)	65 (80.2)	60 (75.0)	12 (85.7)	13 (86.7)	76 (93.8)	71 (88.8)
Serious AEs	6 (7.4)	4 (4.9)	4 (5.0)	0	2 (13.3)	8 (9.9)	5 (6.3)
AEs leading to permanent treatment discontinuation	3 (3.7)	6 (7.4)	7 (8.8)	1 (7.1)	2 (13.3)	11 (13.6)	8 (10.0)
AEs leading to death	0	0	0	0	0	0	0
Most common AEs by system organ class							
Infections and infestations	23 (28.4)	36 (44.4)	24 (30.0)	9 (64.3)	8 (53.3)	55 (67.9)	42 (52.5)
Nasopharyngitis	12 (14.8)	16 (19.8)	12 (15.0)	5 (35.7)	4 (26.7)	27 (33.3)	23 (28.8)
Upper respiratory tract infection	4 (4.9)	6 (7.4)	4 (5.0)	0	0	8 (9.9)	7 (8.8)
Gastroenteritis	1 (1.2)	1 (1.2)	3 (3.8)	0	0	2 (2.5)	6 (7.5)
Cystitis	1 (1.2)	3 (3.7)	1 (1.3)	0	0	5 (6.2)	1 (1.3)
Blood and lymphatic system disorders	0	11 (13.6)	12 (15.0)	2 (14.3)	3 (20.0)	15 (18.5)	13 (16.3)
Neutropenia	0	7 (8.6)	9 (11.3)	2 (14.3)	1 (6.7)	10 (12.3)	9 (11.3)
Vascular disorders	0	3 (3.7)	4 (5.0)	0	1 (6.7)	5 (6.2)	5 (6.3)
Hypertension	0	2 (2.5)	4 (5.0)	0	0	4 (4.9)	5 (6.3)
Gastrointestinal disorders	11 (13.6)	10 (12.3)	20 (25.0)	4 (28.6)	4 (26.7)	19 (23.5)	25 (31.3)
Stomatitis	3 (3.7)	5 (6.2)	8 (10.0)	2 (14.3)	1 (6.7)	6 (7.4)	8 (10.0)
Hepatobiliary disorders	4 (4.9)	9 (11.1)	11 (13.8)	1 (7.1)	2 (13.3)	11 (13.6)	10 (12.5)
Hepatic function abnormal	4 (4.9)	6 (7.4)	8 (10.0)	0	0	8 (9.9)	7 (8.8)
Skin and subcutaneous tissue disorders	8 (9.9)	15 (18.5)	12 (15.0)	1 (7.1)	2 (13.3)	25 (30.9)	19 (23.8)
Eczema	1 (1.2)	4 (4.9)	3 (3.8)	1 (7.1)	0	7 (8.6)	4 (5.0)
General disorders and administration site conditions	3 (3.7)	11 (13.6)	12 (15.0)	1 (7.1)	0	16 (19.8)	15 (18.8)
Injection site erythema	0	7 (8.6)	7 (8.8)	1 (7.1)	0	8 (9.9)	7 (8.8)
Injection site pruritus	0	4 (4.9)	4 (5.0)	1 (7.1)	0	5 (6.2)	4 (5.0)
Investigations	6 (7.4)	11 (13.6)	12 (15.0)	4 (28.6)	0	14 (17.3)	14 (17.5)
ALT increased	4 (4.9)	5 (6.2)	3 (3.8)	3 (21.4)	0	7 (8.6)	4 (5.0)

Values are the number (%) of patients with AEs overall or by system organ class and preferred term (≥ 5 patients by preferred term in any treatment group)

^a^Treatment-emergent AEs during active dose only

*AE* adverse event, *ALT* alanine aminotransferase, *MTX* methotrexate, *q2w* every 2 weeks

Infections were the most common TEAEs in all treatment groups and the most common serious AEs in the active treatment groups. Serious infections were reported in five patients in the 150-mg dose group (herpes zoster, infective myositis, pharyngeal abscess, *Pneumocystis jirovecii* pneumonia, and sepsis) and by one patient in the placebo to 200-mg dose group (*Pneumocystis jirovecii* pneumonia). Opportunistic infections were reported by one patient in the 150-mg dose group and one in the placebo to sarilumab 200 mg group (both *Pneumocystis jirovecii* pneumonia); there were no cases of tuberculosis. Of the six patients reporting serious infections, four (three sarilumab 150 mg and one placebo to sarilumab 200 mg) had an absolute neutrophil count (ANC) ≥ lower limit of normal (LLN) during the study. One patient (sarilumab 150 mg) had a serious infection (localized herpes) concurrent with ANC < LLN (ANC 0.97 Giga/l). Infection led to permanent treatment discontinuation in nine patients: six in the sarilumab 150-mg dose group, one in the 200-mg dose group, and one in each of the placebo to sarilumab groups. Infections were generally not associated with neutropenia, and no increased risk of infection was associated with decreased ANC < 1.0 Giga/l. Most cases of decreases in ANC were to ANC ≥ 1.0 Giga/l (grade 1–2 neutropenia, occurring in 34 (42.0%) and 37 (46.3%) of patients in the 150- and 200-mg groups, respectively; Table 4). ANC < 1.0 Giga/l occurred in 11 (13.6%) patients in the 150-mg group and 6 (7.5%) patients in the 200-mg group.

**Table 4:** Laboratory values through week 52 (safety population)

	Sarilumab			
	Placebo + MTX to 150 mg q2w (*n* = 14)	Placebo + MTX to 200 mg q2w (*n* = 15)	150 mg q2w + MTX (*n* = 81)	200 mg q2w + MTX (*n* = 80)
Absolute neutrophil count, *n* (%)				
Grade 1: ≥ 1.5 Giga/l to < LLN	3 (21.4)	2 (13.3)	14 (17.3)	19 (23.8)
Grade 2: ≥ 1 to < 1.5 Giga/l	2 (14.3)	6 (40.0)	20 (24.7)	18 (22.5)
Grade 3: ≥ 0.5 to < 1 Giga/l	2 (14.3)	0	10 (12.3)	6 (7.5)
Grade 4: < 0.5 Giga/l	1 (7.1)	0	1 (1.2)	0
Hepatic enzyme levels, *n* (%)				
ALT				
> 1 ULN and ≤ 3 ULN	7 (50.0)	10 (66.7)	41 (50.6)	43 (53.8)
> 3 ULN and ≤ 5 ULN	2 (14.3)	0	11 (13.6)	5 (6.3)
> 5 ULN and ≤ 10 ULN	0	1 (6.7)	1 (1.2)	2 (2.5)
> 10 ULN	0	0	0	0
AST				
> 1 ULN and ≤ 3 ULN	9 (64.3)	9 (60.0)	52 (64.2)	42 (52.5)
> 3 ULN and ≤ 5 ULN	1 (7.1)	1 (6.7)	5 (6.2)	2 (2.5)
> 5 ULN and ≤ 10 ULN	0	0	0	1 (1.3)
> 10 ULN	0	0	0	0

The number (*n*) represents the subset of the total number of patients who met the criterion in question at least once during treatment-emergent adverse event period

*ALT* alanine aminotransferase, *AST* aspartate aminotransferase, *LLN* lower limit of normal, *MTX* methotrexate, *q2w* every 2 weeks, *ULN* upper limit of normal

Thrombocytopenia was reported for four patients in the sarilumab 150 mg group and five in the sarilumab 200 mg group. Hepatic disorders were reported in three patients in the placebo to sarilumab 150 mg group and two in the placebo to sarilumab 200 mg group. There were no serious AEs of hepatic disorders. In total, six patients (three in each of the sarilumab dose groups) in the sarilumab groups and one patient in the placebo to sarilumab 200 mg group reported hepatic disorders leading to permanent treatment discontinuation. AE reports of hepatic disorders were driven by abnormalities in liver function tests, with no evidence of liver disease or Hy’s law. Most patients across all groups had ALT and aspartate aminotransferase (AST) values ≤ 3× the upper limit of normal (ULN), and there were no ALT or AST values > 10 ULN (Table 4).

Elevations in lipids were reported in two patients in the sarilumab 150 mg group, five in the sarilumab 200 mg group, and one in the placebo to sarilumab 200 mg group. The events were not serious and did not lead to discontinuation of treatment. There were no major adverse cardiovascular events (MACE) reported after week 24. There was a treatment-emergent cardiovascular event, adjudicated by the Cardiovascular Adjudication Committee as “other cardio/cerebrovascular event (nonfatal),” that did not meet the MACE criteria (right iliac vein thrombus at the time of catheter placement) in a patient in the placebo to sarilumab 200 mg group. There was one treatment-emergent MACE (acute anterior myocardial infarction) that occurred in the placebo group before week 24 in a patient who had a medical history of palpitations and hypercholesterolemia and was a smoker. Treatment was interrupted temporarily; the patient was treated and recovered. At least one treatment-emergent hypersensitivity reaction was reported in 19 patients in the sarilumab 150 mg group, 16 patients in the sarilumab 200 mg group, and 1 patient in the placebo to 150 mg group. There were no hypersensitivity events leading to death, no hypersensitivity serious AEs, and no hypersensitivity events leading to permanent treatment discontinuation. One patient in the sarilumab 200 mg group had a TEAE of malignancy (Bowen’s disease: unspecified tumor). There were no malignancy events leading to death or reported as serious, and no events leading to permanent treatment discontinuation. No patient with malignancy was identified in the placebo to sarilumab groups. There were no cases of gastrointestinal perforation.

In the placebo-controlled period of the study up to 24 weeks, 1.2% (1/81), 1.3% (1/80), and 1.2% (1/81) of patients in the sarilumab 150 mg, sarilumab 200 mg, and placebo groups, respectively, exhibited persistent positive response in the antidrug antibody (ADA) assay. Positive responses in the neutralizing antibody assay were detected in 1.3% (1/80) of patients in the sarilumab 200 mg group. Following the placebo-controlled period, one additional patient in the sarilumab 150 mg group and two additional patients in the sarilumab 200 mg group exhibited persistent positive responses, resulting in 52-week rates of persistent positive ADA response of 2.5% in the sarilumab 150 mg group and 3.8% in the sarilumab 200 mg group. Among the patients who switched from placebo to sarilumab at week 24, there were no patients with a persistent positive response in the placebo to sarilumab 150 mg group and one (6.7%) patient with a persistent positive response in the placebo to sarilumab 200 mg group. In the sarilumab groups, a total of 31 hypersensitivity reactions occurred in patients with negative ADA status and four occurred in patients with positive ADA status. In sarilumab groups, reports of lack of efficacy (permanent treatment discontinuation due to lack of efficacy or switching to open-label rescue treatment) were confined to 18 (12%) ADA-negative patients and one (9.1%) ADA-positive patient; reports of loss of efficacy (permanent treatment discontinuation due to lack of efficacy or switching to open-label rescue treatment after achieving ACR50) were confined to four (2.7%) ADA-negative patients.

Other than the laboratory values noted as AEs of special interest and reported above, there were no safety signals in laboratory, vital signs, or ECG evaluations.

## Discussion

The KAKEHASI study was a 52-week, randomized, fixed-dose, parallel-group trial with a 24-week, randomized, double-blind, placebo-controlled period followed by a 28-week extension in which a dose of either 150 or 200 mg of sarilumab was administered SC q2w with MTX as background therapy in Japanese patients with RA and inadequate response to MTX. Improvements with sarilumab + MTX occurred as early as 2 weeks, as shown by CRP inhibition, with clinical efficacy sustained up to 52 weeks of treatment by both doses of sarilumab, with significant improvement in signs, symptoms, and physical function.

The primary endpoint ACR20 response rates at week 24 were superior to placebo in both sarilumab dose groups, a finding consistent with ACR20 response rates in MOBILITY [5]. For the patients originally receiving sarilumab, the ACR20 and ACR50 responses were consistent with the results at week 24, while the ACR70 response was superior to that at week 24.

In the treatment of RA, a rapid response and full suppression of CRP are both key to a good outcome. In our study, from the second week of starting treatment, a higher proportion of patients in the 200 mg q2w group had CRP levels below 0.02 mg/dl compared with the 150 mg q2w group. With tocilizumab, a humanized mouse immunoglobulin G1 monoclonal antibody against the IL-6R, a higher rate of DAS28-ESR remission and improvement of swollen and tender joint counts after both 24 and 52 weeks was reported among Japanese patients with RA whose CRP levels normalized within 12 weeks of starting treatment compared with those whose levels did not normalize [11].

In the KAKEHASI study, for both groups originally receiving sarilumab + MTX, the exploratory efficacy parameters at week 52 were generally similar between each dose group and generally consistent with the results at week 24. For both groups switching to sarilumab + MTX from placebo + MTX at week 24, the exploratory efficacy parameters were generally similar between each dose group and showed improvements in measures of clinical response. After 12 weeks of treatment, a greater proportion of patients had better control of the signs and symptoms of RA (ACR50 and ACR70) and reduction of disease activity (DAS28-CRP < 2.6, SDAI ≤ 3.3, and CDAI ≤ 2.8) with sarilumab 200 mg + MTX compared with sarilumab 150 mg + MTX. These results suggest that although the 150-mg dose of sarilumab may be sufficient to provide efficacy in terms of ACR20, the higher 200-mg dose may be required in order to see early remission. Achievement of early remission is vital in the treatment of RA, leading to sustained remission, better structural outcome, and ultimately modifying the course of the disease [12].

The safety profiles of sarilumab 150 mg q2w + MTX and 200 mg q2w + MTX at week 52 were generally similar and consistent with the anticipated effects of IL-6 inhibition and the known safety profile of sarilumab.

The proportions of patients with TEAEs were generally similar within the groups originally receiving sarilumab and within the groups switching to sarilumab from placebo at week 24. Low numbers of patients reported serious AEs and/or AEs leading to discontinuation.

Neutropenia appeared as a laboratory abnormality with little or no clinical consequence, as it was not associated with risk of infection. This lack of relationship between neutropenia and infection was also observed in the global MOBILITY and TARGET studies [5, 9] but is best demonstrated in the MONARCH study, in which treatment with sarilumab led to higher levels of neutropenia than with adalimumab, but infection rates were similar [8].

Patients with clinically relevant thrombocytopenia reported no bleeding event. AE reports of hepatic abnormality were driven by abnormalities in liver function tests, with no evidence of liver disease or Hy’s law. Very low and comparable numbers of patients in both sarilumab groups had positive ADA assay responses. Immunogenicity was not associated with loss or lack of efficacy or safety issues. The types and frequency of AEs were similar in the 24- and 52-week time periods, with infections and infestations being the most frequent by system organ class and nasopharyngitis being the most frequent by preferred term. In both the current Japanese study and other global studies, there were no clinically significant differences in safety profile between the sarilumab 150 and 200 mg q2w groups, and no major problems with tolerance in the 200 mg q2w groups [5, 8, 9].

The results of this study are consistent with the anticipated effects of an IL-6 inhibitor [13, 14] and with the results of sarilumab studies in non-Japanese populations [5, 8, 9]. Indeed, bridging with the MOBILITY study was achieved.

There are some limitations to the study findings. The KAKEHASI study was conducted in Japanese patients who generally had long-term RA, with a mean duration of ~ 8 years. Approximately 30% of the patients had previously been treated with biologic DMARDs but had not been categorized as biologic nonresponders; therefore, the population may not be generalizable to a population of Japanese RA patients characterized by an inadequate response to biologic DMARDs. However, post hoc analysis of ACR20 response rates by prior biologic DMARD use (experienced vs naïve) showed rates of 21/28 (75.0%) versus 34/53 (64.2%) for sarilumab 150 mg, 10/22 (45.5%) versus 36/58 (62.1%) for sarilumab 200 mg, and 3/22 (13.6%) versus 9/59 (15.3%) for placebo, respectively. A recent study has shown that sarilumab 150 and 200 mg q2w + csDMARDs is efficacious in patients with an inadequate response or intolerance to anti-TNF agents [9]. A further limitation is the lack of measurement of radiographic progression in this study; however, in the MOBILITY study, radiographic results after 1 year of follow-up showed that the 200-mg q2w dose of sarilumab provided substantially better inhibition of radiographic progression than the 150-mg q2w dose. More patients in the sarilumab 200 mg q2w group had no progression in modified Sharp/van der Heijde score (55.6% vs 47.8%) [5].

## Conclusion

Adding sarilumab at either 150 mg q2w or 200 mg q2w to MTX provides significant improvements in signs and symptoms and physical function, and an additional treatment for Japanese RA patients with insufficient response to MTX. Efficacy and safety profiles were consistent with those seen in sarilumab studies in non-Japanese populations. Despite the availability of a wide range of treatment options for RA, there remains an unmet need globally for the treatment of patients who are intolerant or refractory to current therapies. These important findings show that a new treatment option that has been assessed globally is also effective for Japanese patients with RA.

## Additional file

Additional file 1: **Table S1.** Summary of clinical response after 4 and 12 weeks of treatment (mITT population plus first 12 weeks of active sarilumab in placebo to 150 mg and placebo to 200 mg switch groups). (DOCX 14 kb)

## Abbreviations

ACR: American College of Rheumatology
ACR20/50/70: American College of Rheumatology 20%/50%/70% improvement criteria
ADA: Antidrug antibody
AE: Adverse event
ALT: Alanine aminotransferase
ANC: Absolute neutrophil count
AST: Aspartate aminotransferase
CDAI: Clinical Disease Activity Index
CRP: C-reactive protein
cs: Conventional synthetic
DAS28: Disease Activity Score 28-joint count
DMARD: Disease-modifying antirheumatic drug
ECG: Electrocardiogram
ESR: Erythrocyte sedimentation rate
EULAR: European League Against Rheumatism
HAQ-DI: Health Assessment Questionnaire-Disability Index
hs: High sensitivity
IL-6: Interleukin-6
IL-6R: Interleukin-6 receptor
LLN: Lower limit of normal
MACE: Major adverse cardiovascular event
MedDRA: Medical Dictionary for Regulatory Activities
mITT: Modified intent-to-treat
MTX: Methotrexate
q2w: Every 2 weeks
RA: Rheumatoid arthritis
SC: Subcutaneous
SDAI: Simplified Disease Activity Index
SJC: Swollen joint count
TEAE: Treatment-emergent adverse event
TJC: Tender joint count
TNF: Tumor necrosis factor
ULN: Upper limit of normal
