# Correction to: Sarilumab plus methotrexate in patients with active rheumatoid arthritis and inadequate response to methotrexate: results of a randomized, placebo-controlled phase III trial in Japan

**DOI:** 10.1186/s13075-019-1887-x

**Published:** 2019-04-16

**Authors:** Yoshiya Tanaka, Kazuteru Wada, Yoshinori Takahashi, Owen Hagino, Hubert van Hoogstraten, Neil M. H. Graham, Hideto Kameda

**Affiliations:** 1 The First Department of Internal Medicine, School of Medicine, University of Occupational and Environmental Health, Japan, 1-1 Iseigaoka, Yahata-nishi-ku, Kitakyushu, 807-8555 Japan; 2 Sanofi K.K, Opera City Tower 3-20-2 Nishi-Shinjuku, Shinjuku-ku, Tokyo, 163-1488 Japan; 3 Sanofi, 55 Corporate Drive, Bridgewater, NJ 08807 USA; 4 Sanofi-Genzyme, 500 Kendall St, Cambridge, MA 02142 USA; 5 Regeneron Pharmaceuticals, Inc., 777 Old Saw Mill River Road, Tarrytown, NY 10591 USA; 6 Division of Rheumatology, Department of Internal Medicine, School of Medicine, Toho University, 2-22-36 Ohashi, Meguro-ku, Tokyo, 153-8515 Japan

**Correction to: Arthritis Res Ther**

**https://doi.org/10.1186/s13075-019-1856-4**

Following publication of the original article [1], the authors reported an error in Table 2. The data for ‘HAQ-DI response (MCID ≥ 0.3), n (%) / At week 52’ for the ‘Sarilumab 150 mg q2w + MTX’ and ‘Sarilumab 200 mg q2w + MTX’ groups should be 41 (50.6) and 37 (46.3), respectively (last row, last two entries of the table).

The corrected table is given below.

**Table 2:** Efficacy results (mITT population)

	Sarilumab			
	Placebo to 150 mg q2w + MTX (*n* = 41 (*n* = 14 at week 52))^a^	Placebo to 200 mg q2w + MTX (*n* = 40 (*n* = 15 at week 52))^a^	150 mg q2w + MTX (*n* = 81)	200 mg q2w + MTX (*n* = 80)
Signs and symptoms				
ACR20 response, *n* (%)				
At week 12	15 (18.5)		54 (66.7)^***^	52 (65.0)^***^
At week 24	12 (14.8)		55 (67.9)^***^	46 (57.5)^***^
At week 52	9 (64.3)	10 (66.7)	58 (71.6)	48 (60.0)
ACR50 response, *n* (%)				
At week 12	5 (6.2)		22 (27.2)^***^	25 (31.3)^***^
At week 24	8 (9.9)		35 (43.2)^***^	31 (38.8)^***^
At week 52	8 (57.1)	10 (66.7)	37 (45.7)	38 (47.5)
ACR70 response, *n* (%)				
At week 12	1 (1.2)		5 (6.2)	15 (18.8)^***^
At week 24	3 (3.7)		15 (18.5)^**^	12 (15.0)^*^
At week 52	4 (28.6)	3 (20.0)	29 (35.8)	22 (27.5)
ACR components, mean (SD) change from baseline at week 24				
Tender joint count	− 9.1 (10.2)		− 13.4 (9.9)	− 12.4 (11.3)
Swollen joint count	− 7.2 (6.7)		− 10.6 (8.1)	− 9.5 (9.1)
Pain VAS	− 22.9 (27.7)		− 36.5 (23.4)	− 30.2 (23.3)
Physician global VAS	− 26.8 (18.4)		− 41.8 (21.6)	− 43.9 (19.4)
Patient global VAS	− 18.3 (22.6)		− 32.4 (21.0)	− 30.6 (21.9)
HAQ-DI	− 0.3 (0.4)		− 0.5 (0.5)	− 0.6 (0.5)
CRP, mg/l	− 1.7 (12.2)		− 21.1 (19.5)	− 21.3 (18.0)
DAS28-CRP response, mean (SD) change from baseline				
At week 12	− 0.8 (1.1)		− 2.3 (1.1)^***^	− 2.3 (1.2)^***^
At week 24	− 1.5 (1.2)		− 2.8 (1.0)^***^	− 2.8 (1.1)^***^
At week 52	− 3.1 (1.2)	− 2.9 (1.2)	− 3.2 (1.2)	− 3.2 (1.1)
DAS28-CRP < 2.6, *n* (%)				
At week 12	3 (3.7)		21 (25.9)^***^	27 (33.8)^***^
At week 24	6 (7.4)		29 (35.8)^***^	32 (40.0)^***^
At week 52	7 (50.0)	9 (60.0)	41 (50.6)	43 (53.8)
SDAI, mean (SD) change from baseline				
At week 12	− 8.9 (12.0)		− 20.7 (11.0)^***^	− 18.9 (11.6)^***^
At week 24	− 16.0 (11.6)		− 25.2 (11.6)^***^	− 23.8 (11.3)^***^
At week 52	− 29.6 (9.9)	− 23.4 (12.4)	− 29.4 (13.6)	− 26.9 (11.5)
SDAI ≤ 3.3, *n* (%)				
At week 12	0		2 (2.5)	7 (8.8)^**^
At week 24	1 (1.2)		5 (6.2)	10 (12.5)^**^
At week 52	2 (14.3)	1 (6.7)	19 (23.5)	18 (22.5)
CDAI, mean (SD) change from baseline				
At week 12	− 8.7 (11.4)		− 18.8 (10.6)^***^	− 16.8 (10.9)^***^
At week 24	− 15.7 (11.1)		− 23.1 (11.2)^***^	− 21.7 (10.7)^***^
At week 52	− 28.4 (9.7)	− 21.1 (11.4)	− 27.2 (13.1)	− 24.8 (10.8)
CDAI ≤ 2.8, *n* (%)				
At week 12	0		1 (1.2)	5 (6.3)^*^
At week 24	1 (1.2)		5 (6.2)	8 (10.0)^*^
At week 52	1 (7.1)	0	17 (21.0)	15 (18.8)
Physical function				
HAQ-DI, mean (SD) change from baseline				
At week 12	− 0.1 (0.3)		− 0.4 (0.5)^***^	− 0.4 (0.5)^***^
At week 24	− 0.3 (0.4)		− 0.5 (0.5)^***^	− 0.6 (0.5)^***^
At week 52	− 0.7 (0.6)	− 0.5 (0.3)	− 0.6 (0.6)	− 0.6 (0.6)
HAQ-DI response (MCID ≥ 0.3), *n* (%)				
At week 12	19 (23.5)		39 (48.1)^**^	38 (47.5)^**^
At week 16	19 (23.5)		37 (45.7)^**^	37 (46.3)^**^
At week 24	10 (12.3)		39 (48.1)^***^	39 (48.8)^***^
At week 52	9 (64.3)	8 (53.3)	41 (50.6)	37 (46.3)

*p <  0.05; **p < 0.01; ***p < 0.001

^*a*^Data for combined placebo groups (*n*=81) shown at weeks 12, 16 and 24. ACR American College of Rheumatology, *ACR20/50/70* American College of Rheumatology 20%/50%/70% improvement criteria, *CDAI* Clinical Disease Activity Index, *CRP* C-reactive protein, *DAS28* Disease Activity Score 28-joint count, *HAQ-DI* Health Assessment Questionnaire-Disability Index, *MCID* minimum clinically important difference, *mITT* modified intent-to-treat, *MTX* methotrexate, *q2w* every 2 weeks, *SDAI* Simplified Disease Activity Index, *SD* standard deviation, *SJC* swollen joint count, *TJC* tender joint count, *VAS* visual analog scale
