# Supplementary material for: Sarilumab plus methotrexate in patients with active rheumatoid arthritis and inadequate response to methotrexate: results of a randomized, placebo-controlled phase III trial in Japan
Source: Arthritis Res Ther. 2019 Mar 20;21:79. doi: 10.1186/s13075-019-1856-4 (PMC6425658; doi:10.1186/s13075-019-1856-4)
Supplement: Supplementary file 1 — Table S1. Summary of clinical response after 4 and 12 weeks of treatment (mITT population plus first 12 weeks of active sarilumab in placebo to 150 mg and placebo to 200 mg switch groups). (DOCX 14 kb) [file 13075_2019_1856_MOESM1_ESM.docx]

**Table S1.** Summary of clinical response after 4 and 12 weeks of treatment (mITT population plus first 12 weeks of active sarilumab in placebo to 150 mg and placebo to 200 mg switch groups)

|  | Sarilumab | | | |
| --- | --- | --- | --- | --- |
|  | 150 mg q2w (N=95) | | 200 mg q2w (N=95) | |
|  | Week 4^a^ | Week 12^a^ | Week 4^a^ | Week 12^a^ |
| ACR20 response, n (%) | 35 (36.8) | 65 (68.4) | 42 (44.2) | 64 (67.4) |
| ACR50 response, n (%) | 10 (10.5) | 28 (29.5) | 14 (14.7) | 35 (36.8) |
| ACR70 response, n (%) | 3 (3.2) | 9 (9.5) | 5 (5.3) | 22 (23.2) |
| DAS28-CRP < 2.6 | 11 (11.6) | 29 (30.5) | 14 (14.7) | 37 (38.9) |
| DAS28-CRP low disease activity (< 3.2) | 19 (20.0) | 50 (61.7) | 36 (37.9) | 47 (58.8) |
| SDAI ≤ 3.3 | 1 (1.1) | 21 (25.9) | 2 (2.1) | 27 (33.8) |
| CDAI ≤ 2.8 | 1 (1.1) | 2 (2.5) | 2 (2.1) | 7 (8.8) |

^a^For patients switching from placebo to sarilumab, the data at visits 13 (week 28) and 17 (week 36) were pooled with weeks 4 and 12, respectively, considering the time from start of sarilumab
*ACR20/50/70* American College of Rheumatology 20%/50%/70% improvement criteria, *CDAI* Clinical Disease Activity Index, *CRP* C-reactive protein, *DAS28* Disease Activity Score 28-joint count, *mITT* modified intent-to-treat, *q2w* every 2 weeks, *SDAI* Simplified Disease Activity Index
